# Grape Seed Proanthocyanidin Extract Ameliorates Cardiac Remodelling After Myocardial Infarction Through PI3K/AKT Pathway in Mice

**DOI:** 10.3389/fphar.2020.585984

**Published:** 2020-12-04

**Authors:** Yongxue Ruan, Qike Jin, Jingjing Zeng, Fangfang Ren, Zuoyi Xie, Kangting Ji, Lianpin Wu, Jingguo Wu, Lei Li

**Affiliations:** ^1^ Department of Cardiology, The Second Affiliated Hospital and Yuying Children’s Hospital of Wenzhou Medical University, Wenzhou, Zhejiang, China; ^2^ Department of General Internal Medicine, The First Affiliated Hospital of Sun Yat-sen University, Guangzhou, China; ^3^ Department of Emergency, The First Affiliated Hospital, Sun Yat-Sen University, Guangzhou, China

**Keywords:** grape seed proanthocyanidin extract, myocardial infarction, pi3k/akt pathway, cardiac function, myocardial fibrosis, apoptosis

## Abstract

Myocardial infarction is one of the most serious fatal diseases in the world, which is due to acute occlusion of coronary arteries. Grape seed proanthocyanidin extract (GSPE) is an active compound extracted from grape seeds that has anti-oxidative, anti-inflammatory and anti-tumor pharmacological effects. Natural products are cheap, easy to obtain, widely used and effective. It has been used to treat numerous diseases, such as cancer, brain injury and diabetes complications. However, there are limited studies on its role and associated mechanisms in myocardial infarction in mice. This study showed that GSPE treatment in mice significantly reduced cardiac dysfunction and improved the pathological changes due to MI injury. *In vitro*, GSPE inhibited the apoptosis of H9C2 cells after hypoxia culture, resulting in the expression of Bax decreased and the expression of Bcl-2 increased. The high expression of p-PI3K and p-AKT was detected in MI model *in vivo* and *in vitro*. The use of the specific PI3K/AKT pathway inhibitor LY294002 regressed the cardio-protection of GSPE. Our results showed that GSPE could improve the cardiac dysfunction and remodeling induced by MI and inhibit cardiomyocytes apoptosis in hypoxic conditions through the PI3K/AKT signaling pathway.

## Introduction

Pathologically, Myocardial infarction (MI) is defined as myocardial cell death caused by long-term ischemia (Jennings and Ganote, 1974). Currently, in medicine, the clinical definition of MI refers to the detection of abnormal cardiac biomarkers in the presence of acute myocardial ischemia (Thygesen et al., 2018). Previous studies have shown that myocardial injury, defined by an elevated cardiac troponin value, is frequently encountered clinically and is associated with an adverse prognosis (Sarkisian et al., 2016; Sarkisian et al., 2016). Acute MI is the most serious manifestation of coronary artery disease, causing >4 million deaths in North Asia and Europe (Nichols et al., 2014), and >1/3 of all deaths in developed countries every year (Yeh et al., 2010). Recent years, evidence-based treatment and lifestyle changes have significantly reduced the mortality of artery atherosclerosis and coronary heart disease. However, MI has a great impact on people’s health all over the world, affecting >7 million people every year (Reed et al., 2017).

Grape seed proanthocyanidin extract (GSPE) is an active compound extracted from grape seeds (Tu et al., 2019). It is the source of flavane-3-ol compounds, including catechin and epicatechin monomers, and their respective oligomers (Bladé et al., 2010). It exhibits variety of effects such as anti-apoptotic, anti-inflammatory and improve cell metabolism effects (Sanna et al., 2019). It has potential therapeutic capacity in the treatment of obesity, metabolic syndrome, cancer, diabetes complications and brain injury (Gonzalez-Abuin et al., 2015; Sherif et al., 2017; Gao et al., 2018; Guo et al., 2018). Furthermore, one study showed that GSPE has a potential preventive effect on human colon dysfunction (González-Quilen et al., 2020). The pharmacological effect of GSPE in the heart is mostly associated with cardiac ischemia-reperfusion disease. Some studies have shown that a low dose of GSPE can reduce myocardial cell injury by scavenging ROS, moderately increasing NO production and not inducing cytotoxicity (Rice-Evans, 2004; Shao et al., 2006). In a model of acute oxidative stress induced by ischemia-reperfusion, acute GSPE could protect myocardial cells from I/R injury by activating AKT and producing NO during reperfusion (Shao et al., 2009). There are limited studies on the pharmacological effect of GSPE on MI. Therefore, the aim of this study was to research the pharmacological effect of GSPE on acute MI and its specific signaling pathway.

The PI3K/AKT signaling pathway is a conservative signaling pathway. It can control the response of cells to external stimuli mediated by receptor tyrosine kinases (Li et al., 2014). It coordinates a variety of intracellular signals, and controls cell survival, proliferation and metabolism (Miki et al., 2007; Song et al., 2009). LY 294002 is an inhibitor of PI3K, which can reduce the phosphorylation of downstream AKT, thus blocking the PI3K/AKT signaling pathway (Mocanu et al., 2002; Breivik et al., 2011). Bcl-2 and Bax proteins are two significant members of the Bcl-2 multigene family. It has been found that Bcl-2 has anti apoptotic effects and Bax has pro-apoptotic effects (Adams and Cory, 1998). Numerous researches have shown that AKT protein is a major regulator involved in the transcriptional regulation of Bcl-2 (Ghosh et al., 2013; Chen et al., 2020; Zhang et al., 2020). AKT activation increases the expression of the Bcl-2 through phosphorylation of cyclic AMP response binding protein (Pugazhenthi et al., 2000).

We studied the effects of GSPE on cardiac function and myocardial pathology in mice with MI, as well as the effects of GSPE on the apoptosis of H9C2 cells in a hypoxic environment. In addition, LY294002 was used to further test and verify the activation of PI3K/AKT signaling pathway in MI mice treated with GSPE.

## Materials and Methods

### Animals

C57BL/6J male mice (aged 6 weeks and weighing 20–25 g) were purchased from Beijing Weitong Lihua Experimental Animal Technology Co., Ltd. All mice were placed in a light cycle (half day light and dark) and a temperature of 25 ± 1°C and a humidity of 55 ± 5%, and were allowed free access to water and food. The animals used in the present study were treated in accordance with the Animal Center Guide for the Care and Use of Laboratory Animals. The experimental protocols were approved by the Animal Ethics Committee of the Laboratory Animal Center of Wenzhou Medical University (approval no. wydw2014-0058).

### Drugs and Reagents

GSPE (purity, 95%) was purchased from Tianjin Jianfeng Natural Product R&D Co., Ltd. GAPDH (5174) was purchased from Cell Signaling Technology, Inc. Primary antibodies against phosphorylated PI3K(C73F8), AKT(C67E7), p-AKT (Ser473), Bcl-2 (D17C4), Bax (2772) and a goat anti‐rabbit secondary antibody (4412) were purchased from Cell Signaling Technology, Inc. Primary antibody against p-PI3K (ab182651) and collagen type Ⅲ polyclonal antibody (ab6310) and collagen type Ⅰ antibody (ab34710) were purchased from Abcam (United Kingdom). Anti-α-SMA (13548-1-AP) antibody were purchased from ProteinTech Group, Inc. Cell Counting Kit-8 (CCK-8), SOD and MDA detection kit were purchased from Nanjing Jiancheng Bioengineering Institute. Annexin V-FITC/PI apoptosis detection kit was purchased from Beyotime Institute of Biotechnology.

### Mouse Model of Myocardial Infarction

Mice were anesthetized with 1.5% isoflurane. Mice were intubated and connected to a ventilator to maintain normal breathing. Their heart was then exposed through a lateral incision along the upper edge of the third or fourth rib. The coronary artery of LAD was ligated with a 7-0 polypropylene suture, ∼2–3 mm from the lower edge of the left auricle. The chest was sutured with surgical suture. The sham operation method was the same, except for the LAD coronary artery, which was not ligated.

The mice were randomly divided into five groups, including 1) a sham-operated group (daily oral gavage by 0.2 ml 0.9% normal saline); 2) a GSPE group (daily oral gavage by 0.2 ml GSPE (200 mg/kg) from sham operation to 14 days after operation) (Shi et al., 2019); 3) an MI group (daily oral gavage by 0.2 ml 0.9% normal saline from operation to 14 days after operation); 4) an MI + GSPE-treated group (daily oral gavage by 0.2 ml GSPE (200 mg/kg) from operation to 14 days after operation); and 5) an MI + GSPE + LY group (daily oral gavage by 0.2 ml GSPE (200 mg/kg) and LY294002 (0.2 mg/mouse) from operation to 14 days after operation).

### Doppler Echocardiography Study

Echocardiography was performed in the laboratory by a single experienced echocardiologist. Mice were anesthetized with 1.5% isoflurane and connected to a ventilator to maintain normal breathing. Then, an M-mode transducer (Acuson Sequoia 512; Sonos) was used to performed the transthoracic echocardiography. At the papillary level, the Simpson method was used to measure several main cardiac function indexes (Johri et al., 2011), including the left ventricular ejection fraction (LVEF), the ejection fraction (FS), the left ventricular end diastolic diameter (LVIDd, mm) and the left ventricular end systolic diameter (LVIDs, mm).

### Determination of Myocardial Infarct Size

The 2,3,5-triphenyltetrazolium chloride solution (TTC) staining method was used to determine the myocardial infarct size. 14 days after modeling, euthanasia was done on the mice by intraperitoneal injection of excessive pentobarbital sodium. The hearts of mice were removed quickly and separated from extra connective tissue. The heart was washed with normal saline and frozen at −80°C for 4 h. Five 1–2 mm-thick heart sections were prepared and incubated at 37°C in 2% TTC for 15 min. Next, according to the computer plane measurement, the area of the infarcted tissue was photographed with a digital camera. The infarct area was expressed as the percentage of infarcted area to the risk area x 100%.

### Myocardial Histopathology

In order to evaluate the morphological changes and degree of myocardial fibrosis 14 days after MI, the heart tissues of each group were washed with PBS and fixed with 4% paraformaldehyde solution overnight at 4°C. After the heart tissue was paraffin embedded, the heart was sliced to 5 μm-thick sections. After dehydration and dewaxing of slices, HE staining as well as Masson’s trichrome stain was performed according to the staining kit instructions, followed by observation under a fluorescence microscope (Leica Microsystems GmbH).

### Immunofluorescence Staining

Frozen tissue sections were placed at room temperature for >30 min. After washing three times with PBS, the antigen was blocked with 2% BSA. The tissues were then incubated with anti-α-SMA antibody (1:200) at 4°C for 24 h. The next day, the tissues were incubated at 37°C for 60 min in the dark with a goat secondary antibody (1,500). After three washes with PBS, the tissues were re-stained with DAPI by incubation in the dark for 5 min. Images were obtained with a fluorescence microscope (Leica Microsystems GmbH) and analyzed with Image Proplus 6.0 software (Media Control Silver Spring).

### Oxidative Stress Index

After 14 days of MI, 400 µl blood was collected from the abdominal aorta of mice. The blood was centrifuged at 3,000 rpm at 4°C for 20 min, and then the supernatant was carefully collected. The SOD and MDA test kits pursed from Nanjing Jiancheng Bioengineering Institute were wsed to determine the SOD activity and MDA levels.

### Cell Viability

H9C2 cells were cultured in glucose- and serum-free DMEM without penicillin/streptomycin in a hypoxic atmosphere (95% N_2_, 5% CO_2_) at 37°C (An et al., 2019). The cells were then inoculated into 96-well plates, at a density of 5,000 cells per well. The activity of the cells under different periods of hypoxia and GSPE concentration were measured. The cell activity was measured with a CCK-8 detection kit.

### Apoptosis Detection

H9C2 cells in a 6-well plate (1 × 10^5^ cells/well) were treated as described before and collected with 0.25% trypsin. The H9C2 cells (about 100 μl)) were incubated with 5 μl Annexin V-fluorescein isothiocyanate for 5 min, followed by addition of 3 μl disodium propionate iodide and incubation at room temperature in the dark for 15 min. Finally, the apoptosis rate was measured with a flow cytometer (BD AriaIII; BD Biosciences).

### Western Blotting

The animal proteins extracted from the left ventricular myocardium and the proteins in the cells were cleaved and quantified, and then quantified using the Bradford protein assay (Bio-Rad Laboratories). Proteins were separated by SDS-PAGE and transferred to a PVDF membrane. After blocking with 5% fat-free milk at room temperature for 2 h, the membranes were incubated overnight with the corresponding primary antibody at 4°C, including anti-p-PI3K (1, 1,000 dilution), anti-AKT (1, 2,000 dilution), anti-*p*-AKT (1, 1,000 dilution), anti-Bcl-2 (1, 1,000 dilution), anti-Bax (1, 2,000 dilution), anti-Col-1 (1, 1,000 dilution), anti-Col-3 (1, 1,000 dilution), anti-PI3K (1, 1,000 dilution), anti-α-SMA (1, 3,000 dilution), anti-tubulin (1:5,000 dilution) and anti-GAPDH (1:10,000 dilution). After being washed with TBST, the membranes were incubated with secondary antibodies (1:5,000) for 2 h at room temperature and washed again. ChemiDoc™ XRS + System with Image Lab™ Software purchased from Bio-Rad was used to visualize the signals.

### Statistical Analysis

All statistical data were analyzed using GraphPad Prism 5 (GraphPad Software, Inc.). Values were expressed as the mean ± SEM. Differences between two groups were analyzed using Student’s t-test. The significance of the difference between groups were analyzed by one-way analysis of variance (ANOVA) and followed by LSD post hoc least significant difference test. A value of *p* <0.05 was considered statistically significant difference for all analyses.

## Results

### Grape Seed Proanthocyanidin Extract Improves the Survival Rate and Cardiac Function of MI Mice

**FIGURE 1:**
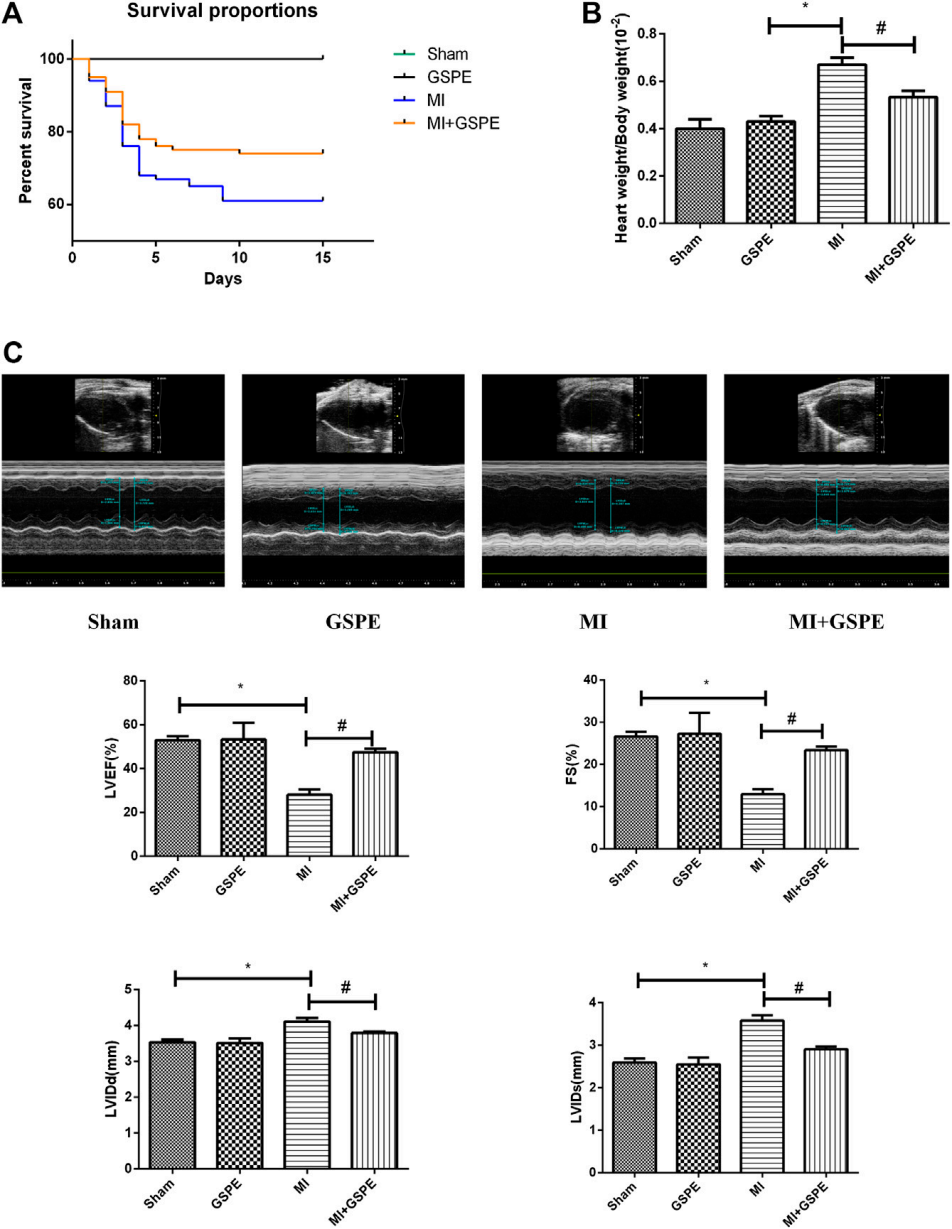
GSPE improved the survival rate and cardiac function of MI mice. **(A)** The survival rate of mice in the GEPE-treated group compared with the MI group (log-rank: *p* < 0.01). (n = 3 per group) **(B)** The heart/body weight ration of mice in different groups. **(C)** Representative M-mode echocardiographic images in short axis from each group, the relative indicators included are LVEF(%), FS(%), LVIDd(mm). Data analyzed are mean ± SD. *Significant difference compared with the control group, *p* < 0.05; significance compared with the MI group, *p* < 0.05. n = 3 per group.

At 14 days post-MI, all mice in the sham group survived, while the survival rate was 61% in the MI group and 74% in the GSPE group (Figure 1A). Compared with that of the MI group, the heart weight/body weight ratio of the GSPE treatment group was significantly lower (Figure 1B). Echocardiography showed that, compared with those in the MI group, the LVEF and FS of the GSPE treatment group were significantly increased, while LVIDd and LVIDs were significantly decreased (Figure 1C).

### Grape Seed Proanthocyanidin Extract Improves Fibrosis in Myocardial Tissue and Myocardial Pathological Changes of MI Mice

**FIGURE 2:**
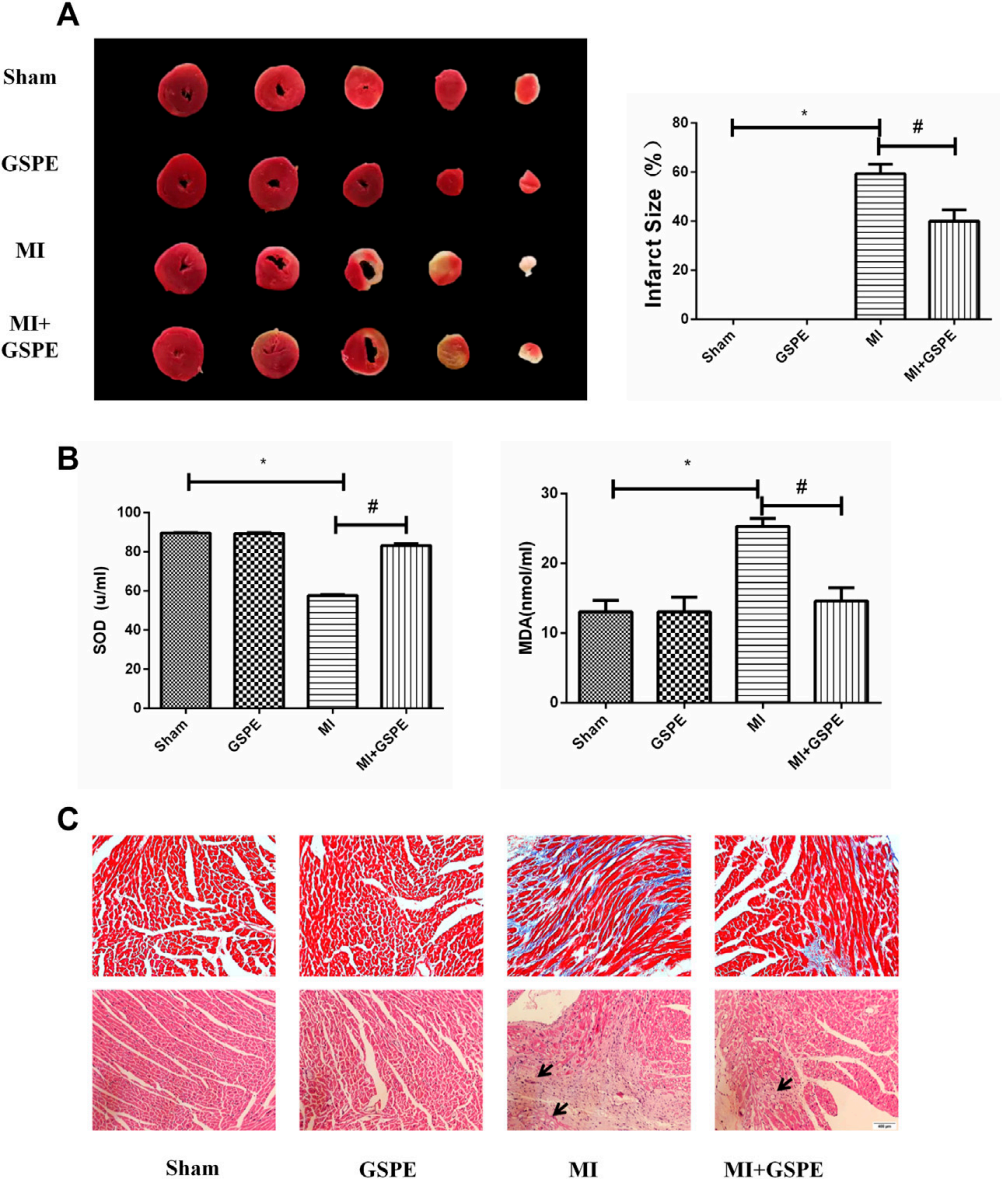
GSPE improved the fibrosis in myocardial tissue and myocardial pathological changes of MI mice. **(A)** Representative image and analysis of infarct size by TTC staining, normal area is red, infarct area is white. **(B)** The contents of SOD and MDA the mice serum in different froup. **(C)** Pathological changes in HE-staining anf Masson staining of cardiac section. The inflammatory cells in HE staining have been marked with arrows. Data analyzed are mean ± SD. *Significant difference compared with the control group, *p* < 0.05; significant difference compared with the MI group, *p* < 0.05. n = 3 per group.

TTC staining showed that the infarct size of the GSPE group was significantly smaller than that of the MI group (Figure 2A). The MDA level in the MI group increased, while GSPE treatment could significantly reduce the increase in MDA. On the contrary, the serum SOD level in the MI group decreased, and GSPE treatment could significantly increase the SOD value (Figure 2B). Masson’s trichrome stain showed that the collagen deposition area of myocardial fibrosis of the MI group was significantly higher than that of MI + GSPE group (Figure 2C). H&E staining showed that myocardial necrosis, inflammatory infiltration and interstitial edema were decreased in the MI + GSPE group compared with those in the MI group (Figure 2C).

### Grape Seed Proanthocyanidin Extract Attenuates MI-Induced Apoptosis and Myocardial Fibrosis

**FIGURE 3:**
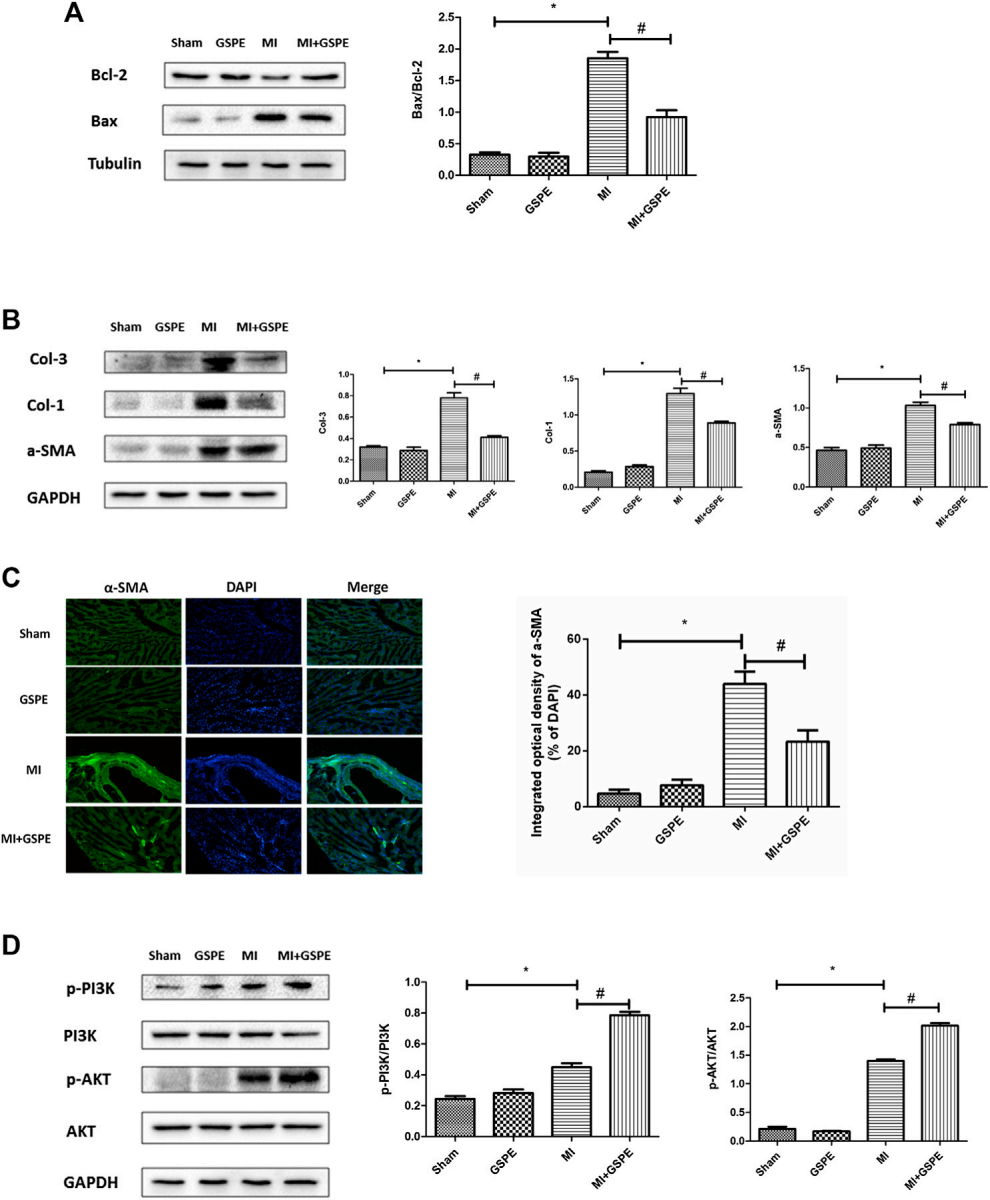
GSPE attenuated MI induced apoptosis and myocardial fibrosis by activating PIK/AKT pathway. **(A)** Effects of GSPE on the levels of Bax and Bcl-2 evaluated by Western blot analysis. **(B)** Effects of GSPE on the levels of Col-1, Col-3 and a-SMA evaluated by Western blot analysis. **(C)** Immunofluorescense staining of a-SMA. **(D)** Effects of GSPE treatment on cardiac levels of p-PI3K, PI3K, p-AKT and AKT evaluated by Western blot analysis. *Significant difference compared with the control group. *p* < 0.05; significant difference compared with the MI group, *p* < 0.05, n = 3 per group.

The Bcl-2 protein family is associated with the regulation of cell apoptosis. In this family, Bcl-2 plays an anti-apoptotic role, while Bax plays a pro-apoptotic role (Bogner et al., 2020). GSPE significantly increased the expression of Bcl-2 and decreased the expression of Bax in myocardial tissue after MI (Figure 3A). In addition, GSPE reduced the expression of collagen Ⅰ, collagen Ⅲ and a-SMA (Figure 3B). Furthermore, immunofluorescence showed that the integral optical density of a-SMA in the GSPE treatment group was significantly lower than that in the MI group (Figure 3C).

### Grape Seed Proanthocyanidin Extract Exerts a Protective Effect on Mouse Heart by Activating the PI3K/AKT Pathway

The PI3K/AKT pathway is one of the important signaling pathways in cells, which participates in the regulation of multiple signaling molecules (Fajgenbaum et al., 2019). Western blotting revealed that p-PI3K and p-AKT expression increasing was detected in the MI group, and GSPE treatment enhanced this activation (Figure 3D).

### Inhibition of the PI3K/AKT Pathway Reverses the Cardiac Protective Effect of GSPE *In Vivo*

**FIGURE 4:**
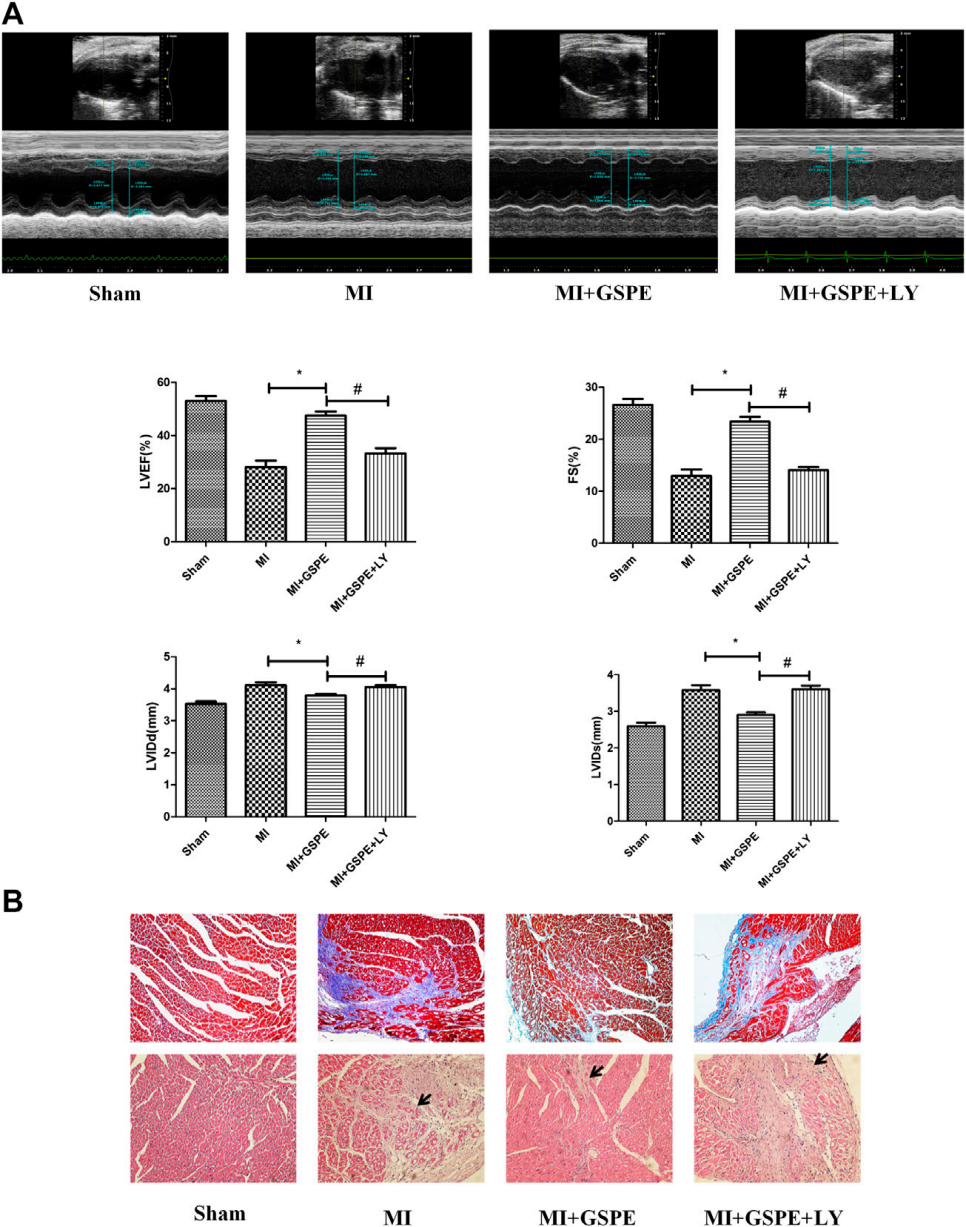
Inhibition of the PI3K/AKT pathway reversed the protective effect of GSPE on cardiac function in vivo. **(A)** Representative M-mode echocardiographic images in short axis from each group, the relative indicatiors included are LVEF(%), FS(%), LVIDd(mm) and LVIDs(mm). **(B)** Pathological changes in HE-staining and Masson staining of cardiac section. The inflammatory cells in HE staining have been marked with arrows. Data analyzed are mean SD. *Significant difference compared with the MI group, *p* < 0.05; significant difference compared with the MI + GSPE group, *p* < 0.05. n = 3 per group.

Echocardiography showed that, compared with those in the MI + GSPE group, the LVEF and FS of the LY294002 treatment group were significantly decreased, while LVIDd and LVIDs were significantly increased (Figure 4A). Masson’s trichrome stain also showed that the collagen deposition area of myocardial fibrosis of the MI + GSPE + LY294002 group was significantly higher than that of MI + GSPE group (Figure 4B). H&E staining showed that myocardial necrosis, inflammatory infiltration and interstitial edema were increased in the MI + GSPE + LY294002 group compared with those in the MI + GSPE group (Figure 4B).

### Inhibition of PI3K/AKT Pathway Reverses the Anti-Apoptosis and Anti-Fibrosis Effects of GSPE

**FIGURE 5:**
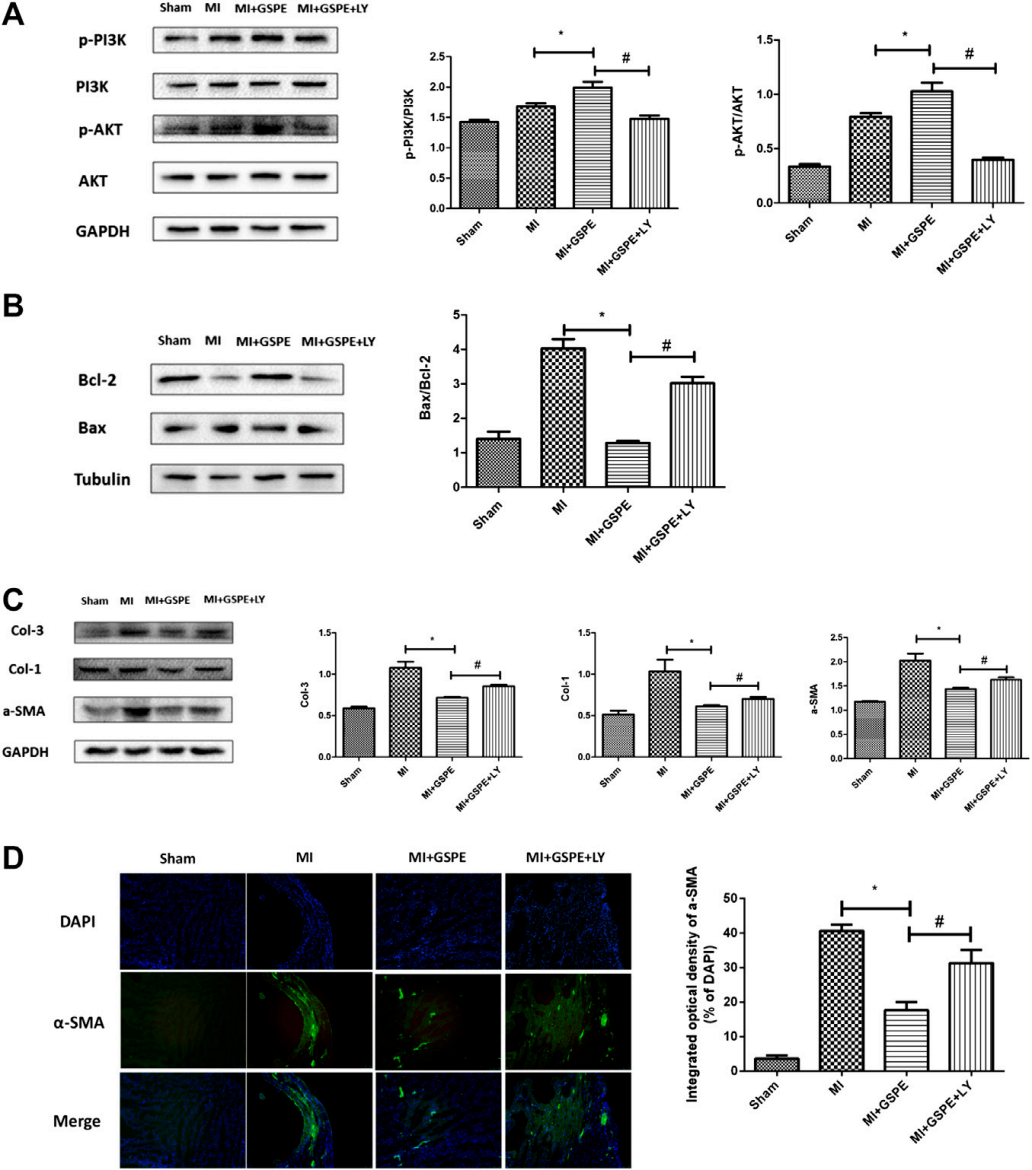
Inhibition of PI3K/AKT pathway can reverse the anti-apoptosis and anti-fibrosis effects of GSPE. **(A)** Effects of GSPE treatment on cardiac levels of p-PI3K, PI3K, p-AKT evaluated by Western blot analysis. **(B)** Effects of GSPE on the levels of Bax and Bcl-2 evaluated by Western blot analysis. **(C)** Effects of GSPE on the levels of Col-1, Col-3 and a-SMA evaluated by Western blot analysis. **(D)** Immunofluorescence staining of a SMS. *Significant difference comapred with the MI froup, *p* < 0.05; significant difference compared with the MI + GSPE group, *p* < 0.05. n = 3 per group.

After administration of the PI3K inhibitor LY294002, the p-PI3K and p-AKT levels in the LY294002-treated group were lower than those in the GSPE group after MI (Figure 5A). Compared with those in the GSPE group, LY294002 increased the level of Bax protein, decreased Bcl-2 levels (Figure 5B), and increased collagen Ⅰ, collagen Ⅲ and α-SMA levels (Figure 5C). In other words, LY294002 significantly increased apoptosis and fibrosis compared with the results of the MI + GSPE group. Furthermore, immunofluorescence showed that the integral optical density of α-SMA in the LY294002 treatment group was higher than that of the MI + GSPE group (Figure 5D).

### Grape Seed Proanthocyanidin Extract Reduces Apoptosis in H9C2 Cells Under Oxygen-Glucose Deprivation

**FIGURE 6:**
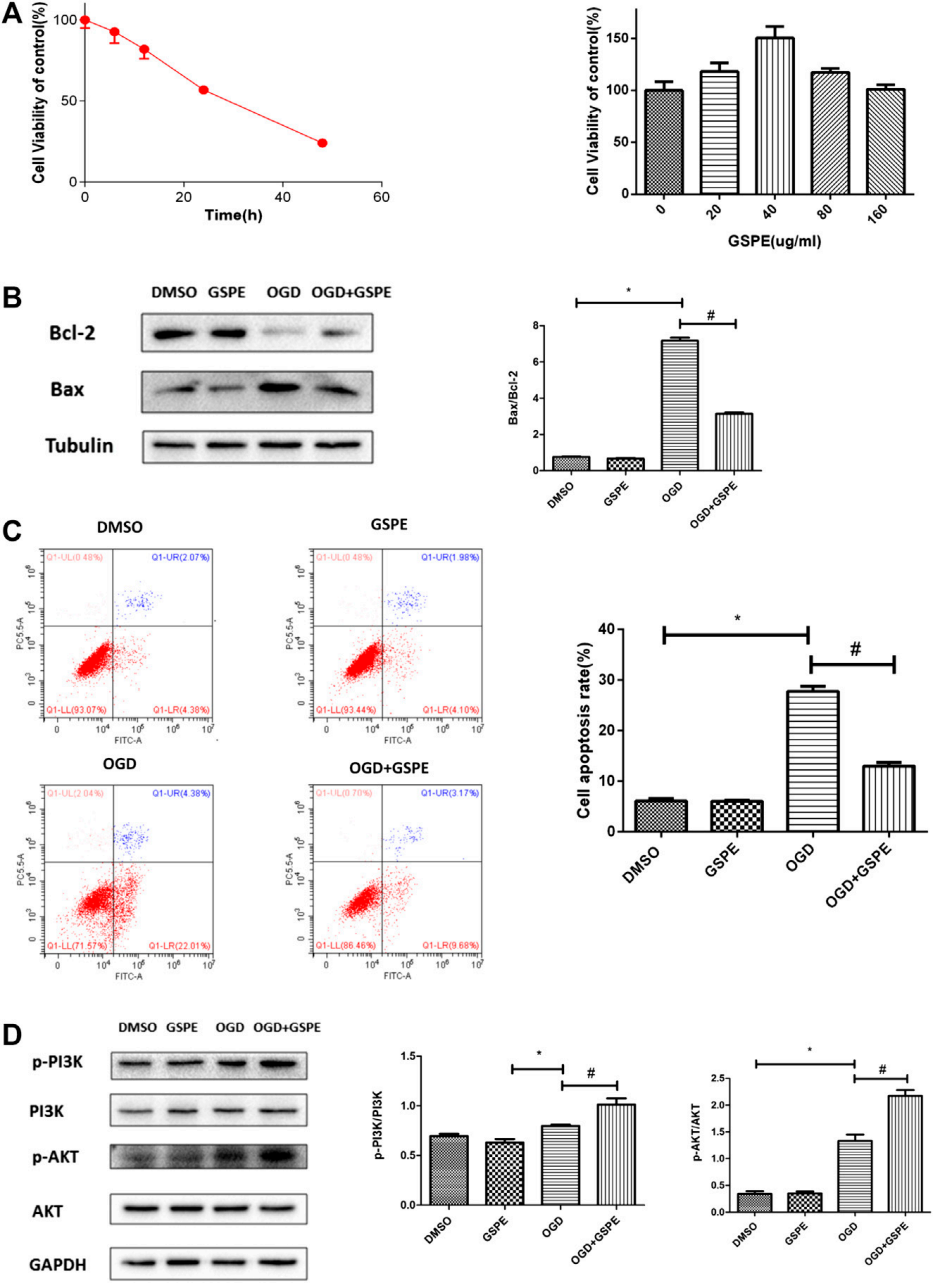
GSPE reduced apoptosis in H9C2 cells under oxygen-glucose deprivation through PI3K/AKT pathway. **(A)** H9C2 cells were culture under hypoxia condition for different time, CCK8 assay was used to assess the cell viability. The appropriate GSPE concentration eas evaluated by CCK8 assay after the cells were cultured under glucose oxygen deptrivation for 24 hours. *Significant difference compared with the DMSO group, P < 0.05. **(B)** Effects of GSPE on the expression of Bax and Bcl-2 evaluated by Western blot analysis. **(C)** H9C2 cells in different treatment were stained by Annexin V-FITC/PI staining and then distinguished by flow cytometer. The cell apoptosis rate was quantified. **(D)** Effects of GSPE treatment on the H9C2 cells of p-PIK, PIK, p-AKT and AKT evaluated by Western blot analysis. Data analyzed are mean ± SD. *Significant difference compared with the DMSO group, *p* < 0.05; significant difference compared with the OGD group, *p* < 0.05. n = 3 per group.

The time-cell activity experiment showed that the most suitable hypoxia time for H9C2 cell apoptosis was ∼24 h (Figure 6A). The dose response experiment showed that the optimal concentration of GSPE was 40 μg/ml (Figure 5A). Western blotting showed that the increasing of Bax under hypoxia, and GSPE treatment significantly decreased the level of Bax (Figure 6B). Furthermore, GSPE treatment increased the expression of Bcl-2 (Figure 6B). In addition, flow cytometry also showed that GSPE could reduce the apoptosis of H9C2 cells under glucose and oxygen deprivation (Figure 6C).

### GSPE Attenuates Cell Apoptosis in H9C2 Cells Under Glucose and Oxygen Deprivation Through the PI3K/AKT Pathway

*In vitro*, western blotting showed that the levels of p-PI3K and p-AKT in the MI + GSPE group were higher than those in the hypoxia group, indicating that GSPE can enhance the activation of the PI3K/AKT pathway in H9c2 cells under hypoxia (Figure 6D).

### Inhibition of the PI3K/AKT Pathway Reverses the Protective Effect of GSPE on H9C2 Cells

**FIGURE 7:**
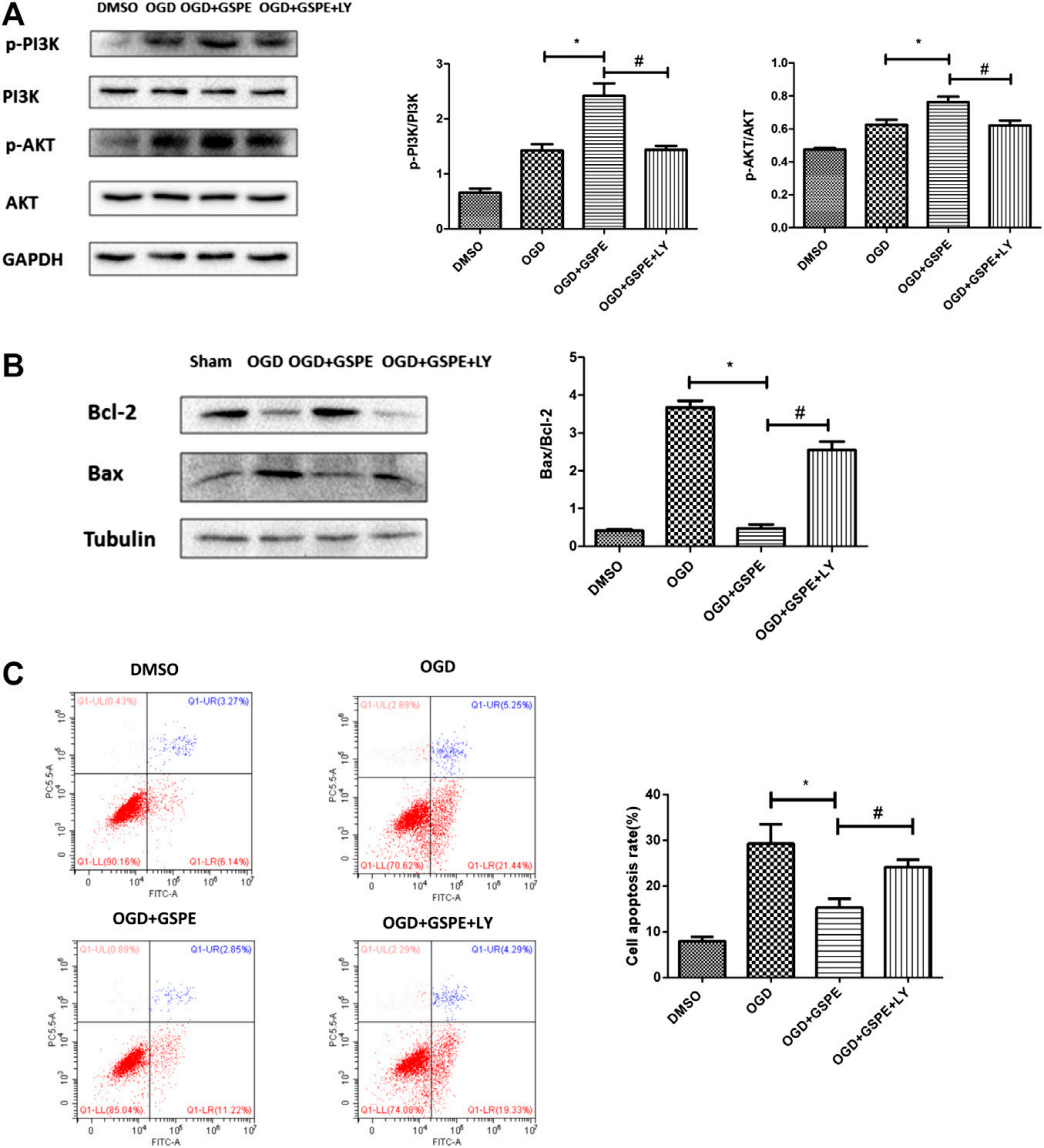
Inhibition of PI3K/AKT pathway reversed the protective effect of GSPE on H9C2 cells. **(A)** Effects of GSPE treatment on the H9C2 cells of p-PI3K, PI3K, p-AKT and AKT evaluated by Western blot analysis. **(B)** Effects of GSPE on the expression of Bax and Bcl-2 evaluated by Western blot analysis. **(C)** H9C2 cells in different treatment were stained by Annexin V-FITC/PI staining and then distinguished by flow cytometer. Then cell apoptosis rate was quantified. *Significant difference compared with the OGD group, *p* < 0.05; significant difference compared with the OGD + GSPE group, *p* < 0.05. *p* = 3 per group.

After administration of the PI3K inhibitor LY294002, the p-PI3K and p-AKT levels in the LY294002 treatment group were lower than those in the GSPE group under OGD (Figure 7A). Compared with those in the GSPE treatment group, the level of Bax increased and the level of Bcl-2 decreased in the LY294002 group (Figure 7B). In addition, flow cytometry showed the same results (Figure 7C). This suggests that GSPE plays an anti-apoptotic role via the PI3K/AKT pathway *in vitro*, which was corroborated by flow cytometry.

## Conclusion

The present study demonstrated that GSPE could improve the cardiac dysfunction and remodeling in mice induced by MI and inhibit cardiomyocytes apoptosis in hypoxic conditions through the PI3K/AKT signaling pathway.

## Discussion

The objective of this research was to find out the pharmacological effects of GSPE on MI in mice. An *in vivo* model of AMI was established by ligating the left anterior descending coronary artery (LAD) of the mouse heart for 14 days (Selvaraju et al., 2020). According to pathological sections and TTC staining of ultrasonic memory heart, the model was successful. In the experiment *in vitro*, the method of glucose and oxygen deprivation was employed to simulate the ischemia and hypoxia of myocardial cells in the infarcted and marginal areas in mice with MI (Abbruzzese et al., 2020). The experimental results showed that intragastric administration of GSPE in mice could reduce heart damage in mice with coronary artery ligation, and the cell experiment produced the same results. Furthermore, the activation of PI3K/AKT signaling pathway was found in the MI mice treated with GSPE. Moreover, by using LY294002, the therapeutic effect of GSPE was reversed, which confirmed the hypothesis. *In vivo* and *in vitro* experiments found that GSPE has a protective effect on short-term MI in mice, and this effect is partly mediated through the activation of the p-PI3K and p-AKT levels.

In a previous study, the PI3K/AKT signaling pathway was one of the main ways to prevent myocardial hypertrophy and apoptosis (Cao et al., 2011). Another study on I/R injury and PI3K signaling showed that activation of the PI3K signaling pathway can reduce the size of MI and significantly improve left ventricular function (Nagaoka et al., 2015). The PI3K/AKT signaling pathway can inhibit the expression of endoplasmic reticulum stress-related proteins after myocardial ischemia-reperfusion, thereby reducing cardiomyocyte apoptosis (Shen et al., 2019). In addition, a study showed that the protective effect of the PI3K/AKT signaling pathway may be associated with the upregulation of Cx43 (Bian et al., 2015). AKT regulates the activity of a variety of downstream molecules, including mammalian target of rapamycin, glycogen synthase kinase 3β and p70S6 kinase, and these proteins can regulate cellular metabolism after combined and phosphorylated (Xu et al., 2016). Several studies have shown that AKT phosphorylation at Ser473 in the PI3K/AKT pathway can partially regulate the expression of Nrf-2, and ultimately increase the activity of SOD and decrease the levels of MDA and ROS (Ma et al., 2014; Liu et al., 2019). In another study of rats MI model, silencing Annexin3 gene and activating of PI3K/AKT signaling pathway can promote the repair and healing of myocardial tissue, which indicates that PI3K/ AKT signaling pathway can accelerate the repair of heart injury (Meng et al., 2019). In a previous study, LY294002 was used to study the myocardial protective role of PI3K/AKT signal in septicemia, and it can induce the expression of Bcl-2 and decrease the expression of Bax, suggesting that it has an inhibitory effect on apoptosis (An et al., 2016). These researches indicate that PI3K/ AKT pathway plays an essential role in cardiac injury and affects the changes of many intracellular substances.

Previous studies have shown that GSPs mainly have antioxidant effects, which can act as antioxidants *in vivo* and directly scavenge ROS, and have been found to have cardioprotective capacity (Sato et al., 1999). One study has shown that GSPE has antioxidant activity in model of myocardial ischemia-reperfusion of mice, and its cardioprotective effect is partly due to its blocking of anti-apoptotic signals by inhibiting JNK-1, c-Jun and other pro-apoptotic transcription factors and genes (Sato et al., 2001). Under the stimulation of GSPE, AKT was activated, and it could activate eNOS and increase the level of NO in primary myocardial cells of chicks in an ischemia/reperfusion model (Shao et al., 2009). These results suggest that the cardioprotective effect of GSPE may be associated with the activation of the PI3K/AKT signaling pathway. The mechanism of GSPE on MI in mice has not been elucidated in previous studies. Therefore, we speculate that the cardio-protective effect of GSPE in MI may be associated with the activation of the PI3K/AKT signaling pathway. In our experiment, the PI3K/AKT signal pathway was activated in MI. GSPE could enhance the activation, and reduce myocardial injury and fibrosis. LY294002, a PI3K inhibitor, partially attenuated the protective effect of GSPE on cardiomyocytes. Therefore, GSPE protects against MI-induced cardiac injury through the PI3K/AKT signal pathway.

There were several limitations in this study. Firstly, whether GSPE could reduce myocardial injury through other signal transduction pathways has not been fully elucidated in this study. We have detected ERK signaling pathway before, and their protein expression has no obvious change before and after GSPE intervention. Secondly, The purity of grape seed cyanidin extract purchased by different pharmaceutical companies is different, and the experimental effect may be varied. Besides, The H9C2 cells are not cardiomyocytes, to validate our findings *in vitro*, primary cardiomyocytes was need. The morphology of neonatal mouse cardiomyocytes was demonstrated in MP4. As presented in the Supplementary Material, we have done some experiments on neonatal mouse cardiomyocytes. GSPE can inhibit neonatal cardiomyocyte apoptosis and affect the expression of apoptotic proteins in the model of glucose and oxygen deprivation. In the future, we will go further in the pharmacological of GSPE on neonatal Cardiomyocyte. We may explore the cell migration, proliferation and chemotaxis effects of GSPE. We will go further into this research in the future.

For the current clinical situation of MI heart disease that cannot be effectively treated, it is necessary to find new and additional drug targets. This research may provide some new ideas for the treatment of myocardial infarction.

## Data Availability Statement

The original contributions presented in the study are included in the article/Supplementary Material, further inquiries can be directed to the corresponding authors.

## Ethics Statement

The animal study was reviewed and approved by Animal Ethics Committee of the Laboratory Animal Center of Wenzhou Medical University (approval no. wydw2014-0058).

## Author Contributions

YR and LL conceived and designed the study. YR performed the experiments. JZ, QJ, FR, and ZX analyzed the data. KJ, LW, and JW contributed reagents and materials. YR wrote the manuscript.

## Funding

This work was supported by National Natural Science Foundation of China (81770292) and Key projects of workstation of He Lin (18331101) and Wenzhou Science and Technology Major Projects (2018ZY007).

## Conflict of Interest

The authors declare that the research was conducted in the absence of any commercial or financial relationships that could be construed as a potential conflict of interest.
