# Supplementary figures and images for: Grape Seed Proanthocyanidin Extract Ameliorates Cardiac Remodelling After Myocardial Infarction Through PI3K/AKT Pathway in Mice
Source: Front Pharmacol. 2020 Dec 4;11:585984. doi: 10.3389/fphar.2020.585984 (PMC7747856; doi:10.3389/fphar.2020.585984)

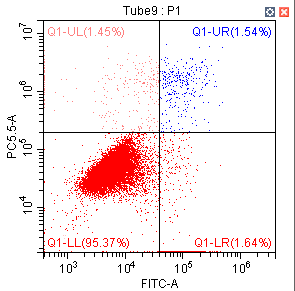

Supplement: Supplementary file 2 [file datasheet1.zip › original file of Supplementary materials/DMSO.gif]

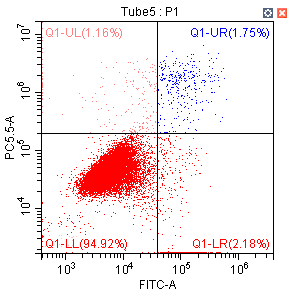

Supplement: Supplementary file 2 [file datasheet1.zip › original file of Supplementary materials/GSPE.gif]

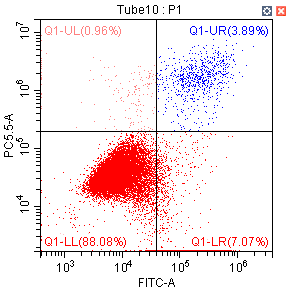

Supplement: Supplementary file 2 [file datasheet1.zip › original file of Supplementary materials/OGD+FSPE.gif]

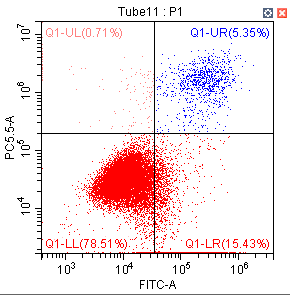

Supplement: Supplementary file 2 [file datasheet1.zip › original file of Supplementary materials/OGD.gif]
