# Supplementary material for: Grape Seed Proanthocyanidin Extract Ameliorates Cardiac Remodelling After Myocardial Infarction Through PI3K/AKT Pathway in Mice
Source: Front Pharmacol. 2020 Dec 4;11:585984. doi: 10.3389/fphar.2020.585984 (PMC7747856; doi:10.3389/fphar.2020.585984)
Supplement: Supplementary file 3 [file datasheet2.docx]

**A**


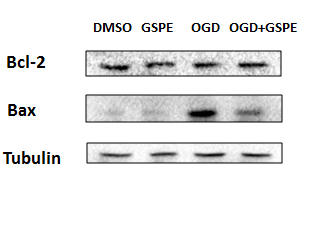

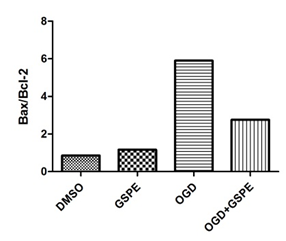


**B**

**GSPE**

**DMSO**


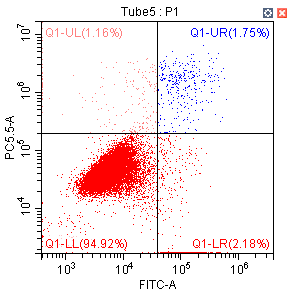

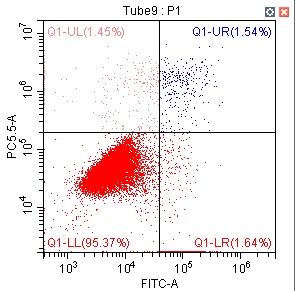


**OGD+GSPE**

**OGD**


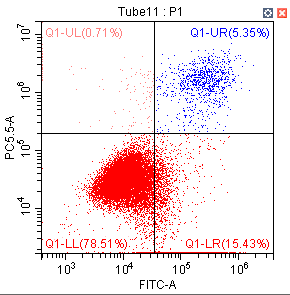

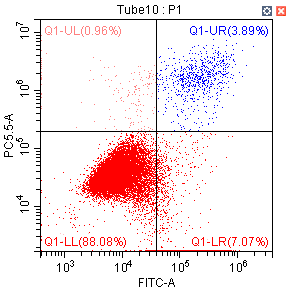


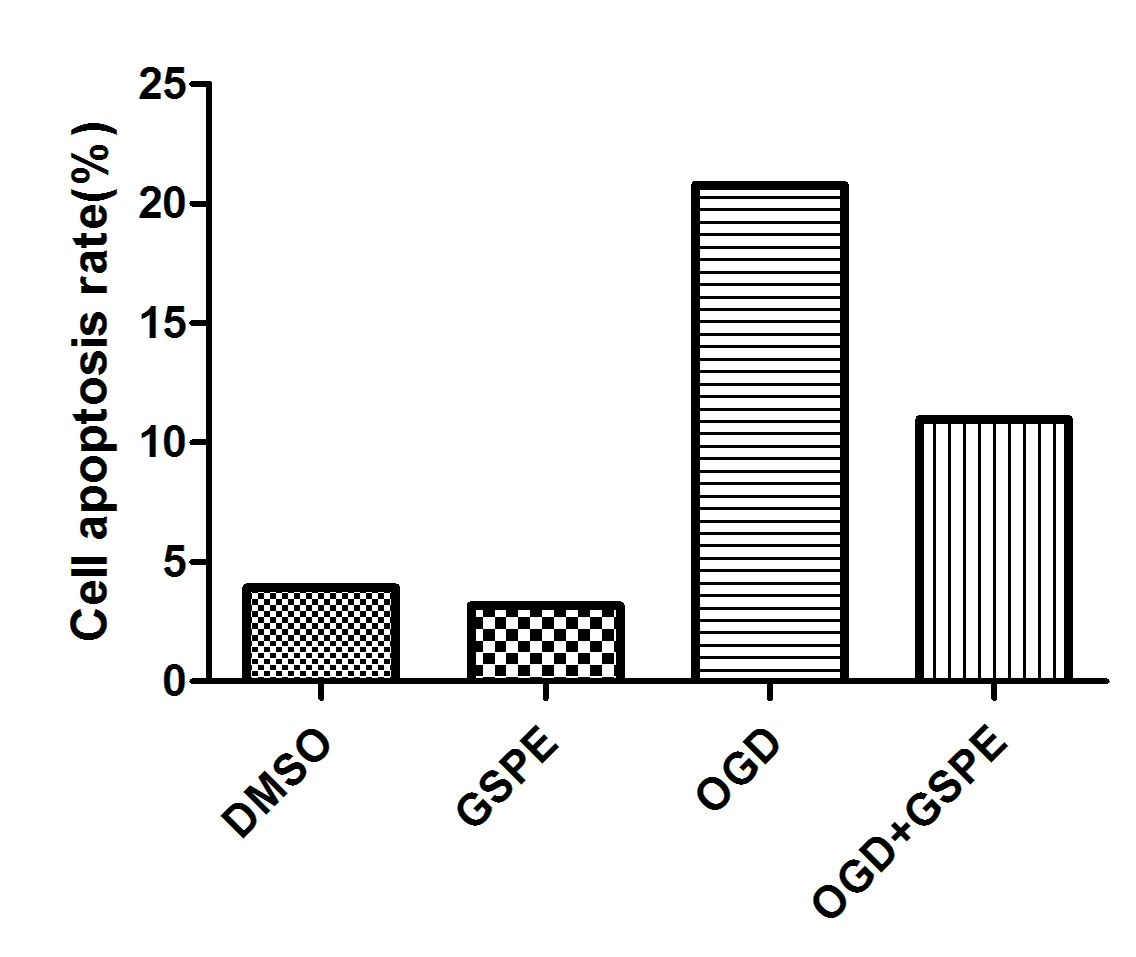


**Supplementary materials | GSPE reduced apoptosis in Neonatal mouse cardiomyocytes under oxygen-glucose deprivation.** (A) Neonatal mouse cardiomyocytes were culture under glucose oxygen deprivation for 24 hours. Then cell apoptosis rate was quantified by Annexin V-FITC/PI staining and then distinguished by flow cytometer. (B) Effects of GSPE on the expression of Bax and Bcl-2 evaluated by Western blot analysis.
